# Antibiotic Prescribing in Hospitalized Pediatric Patients Before Implementation of a Syndromic Antibiogram at Maputo Central Hospital, Mozambique

**DOI:** 10.3390/pediatric18040110

**Published:** 2026-08-10

**Authors:** Darlenne B. Kenga, Jahit Sacarlal, Mohsin Sidat, Valéria Chicamba, Andrea Ntanga Kenga, Yara Manjate, Manuel D. Naiene, Raquel I. Langa, Ramígio Pololo, Troy D. Moon

**Affiliations:** 1 Department of Microbiology, Faculty of Medicine, University Eduardo Mondlane, Maputo C.P. 257, Mozambique; jahityash2002@gmail.com (J.S.); yaramanjate.ym@gmail.com (Y.M.); naiene2000@gmail.com (M.D.N.); raquellanga67@gmail.com (R.I.L.); 2 Department of Community Health, Faculty of Medicine, University Eduardo Mondlane, Maputo C.P. 257, Mozambique; mmsidat@gmail.com; 3 Pediatric Intensive Care Unit, Hospital Central de Maputo, Maputo 1100, Mozambique; vickyy.valeria@gmail.com; 4 National Institute of Health, Marracuene 1120, Mozambique; andrea.kenga@ins.gov.mz (A.N.K.); ramigio.pololo@ins.gov.mz (R.P.); 5 Department of Tropical Medicine and Infectious Diseases, Tulane University Celia Scott Weatherhead School of Public Health and Tropical Medicine, New Orleans, LA 70112, USA; tmoon2@tulane.edu

**Keywords:** antimicrobial resistance, empiric antibiotic prescribing, AWaRe, pediatric patients, antimicrobial stewardship, syndromic antibiogram, Mozambique

## Abstract

**Background:** Empiric antibiotic prescribing is frequently used in low- and middle-income countries because microbiological diagnostic capacity is often limited, highlighting the need for locally generated microbiological data to support evidence-informed empiric antibiotic selection. However, data on pediatric antibiotic prescribing practices in Mozambique remain scarce. This study aimed to characterize empiric antibiotic prescribing among hospitalized pediatric patients at Maputo Central Hospital prior to the implementation of a syndromic antibiogram intervention. **Methods:** An exploratory, retrospective, descriptive baseline analysis was conducted among pediatric patients aged 1 month to 14 years admitted with suspected bacterial infections between January and December 2023, prior to the implementation of a syndromic antibiogram intervention. Sociodemographic, clinical, microbiological, and antibiotic prescribing data were extracted from clinical records. Antibiotics were classified according to the WHO AWaRe framework, and associations between patient characteristics, treatment strategies, and outcomes were analyzed using R software version 4.6.0. **Results:** A total of 358 pediatric patients were included, with a median age of 14 months (IQR: 6–48), and 57% were male. Lower respiratory tract infections were the most frequent diagnosis. Empiric treatment predominated, accounting for 89% of cases, whereas microbiologically guided therapy was observed in only 11%. Urinary tract infections showed significantly higher proportions of microbiologically guided treatment compared with respiratory infections (*p* < 0.001). Monotherapy predominated, while Watch antibiotics accounted for 64.7% of prescriptions. Prolonged hospitalization was associated with respiratory distress, decreased skin turgor, referral status, infection type, and anemia. **Conclusions:** Pediatric antibiotic prescribing was characterized by extensive empiric therapy and high Watch antibiotic use, highlighting important antimicrobial stewardship challenges in Mozambique.

## 1. Introduction

Antimicrobial resistance (AMR) constitutes one of the most significant global public health threats of the 21st century and is estimated to contribute to approximately 700,000 deaths annually [1]. In 2019, approximately 250,000 deaths were directly attributable to AMR in the African region, underscoring the disproportionate burden borne by low- and middle-income countries (LMICs) [2,3].

Antibiotics are among the most commonly used medications in pediatric populations and play a critical role in treating infectious diseases globally [4,5]. While essential for reducing childhood morbidity and mortality, irrational antibiotic use accelerates AMR by selecting for resistant pathogens, leading to higher rates of treatment failure, more severe illness episodes, increased healthcare costs, and elevated mortality [1,6].

In many African settings, limited laboratory capacity and access to appropriate diagnostic tools compel clinicians to rely on empiric antibiotic prescribing based on presumptive clinical diagnoses, a practice that is frequently inappropriate and contributes substantially to the emergence and spread of AMR [2,7,8,9,10,11,12,13]. This problem is exacerbated by a high burden of bacterial infections, fragile healthcare systems, and the absence or underdevelopment of institutional mechanisms, such as infection prevention and control committees and antimicrobial stewardship programs (ASPs). Consequently, clinicians often rely on empiric antibiotic therapy in the absence of readily accessible local microbiological and antimicrobial susceptibility data to inform treatment decisions [2,3,14,15,16].

Point prevalence studies conducted across Africa report that approximately 50% of hospitalized patients receive antibiotics. Prescriptions predominantly involved agents from the World Health Organization (WHO) Access and Watch categories of the Access, Watch, and Reserve (AWaRe) antibiotic classification framework. The most commonly prescribed antibiotics included penicillins, ceftriaxone, gentamicin, and metronidazole, with the highest rates of usage observed in intensive care units (ICUs) and pediatric medical wards [7,9,17,18].

A comparable pattern has been reported in Mozambique, where a very high prevalence of antibiotic use (97.5%) was observed among 315 pediatric inpatients at a referral hospital in the country’s north, for example. Most children were treated for gastroenteritis, pneumonia, or malaria, with ceftriaxone and penicillin being the most commonly prescribed agents; 23.7% of prescriptions fell within the WHO’s Watch category. Antibiotics were predominantly administered parenterally, and most prescriptions were found to contain errors, primarily related to treatment duration and dosing [5,19].

Appropriate prescribing practices depend on accurate identification of the infectious etiology; however, resource limitations frequently lead to antibiotic use in the absence of a confirmed bacterial infection [20,21]. A cross-sectional study conducted in two district hospitals in Maputo Province, in southern Mozambique, found that antibiotic prescribing for pediatric patients was largely driven by clinical presentation and age. Limited access to timely laboratory diagnostic information frequently necessitates empiric antibiotic therapy, underscoring the importance of local microbiological and antimicrobial susceptibility data to support evidence-informed prescribing [20].

Rational antibiotic prescribing should be individualized, taking into account the clinical diagnosis, as well as the patient’s age, sex, weight, organ function, microorganism susceptibility, potential drug and drug–food interactions, and socioeconomic context [1]. In addition, the WHO AWaRe antibiotic classification framework, aligned with the WHO Model Lists of Essential Medicines, serves as an essential tool for optimizing and monitoring the quality and quantity of antibiotic use in the empiric treatment of common diseases in both children and adults, thereby supporting global efforts to combat AMR [16,19,22].

Data on antibiotic prescribing patterns among hospitalized children in Mozambique remain limited. Results presented here are part of a larger implementation science study evaluating the introduction of a syndromic antibiogram intervention within the pediatric wards of Maputo Central Hospital (*Hospital Central de Maputo, HCM*), with the objective of informing the development of effective strategies for the intervention’s future adoption. Our protocol has been published elsewhere [23]. The intervention was designed to support empiric antibiotic prescribing for hospitalized pediatric patients admitted with suspected bacterial infections across multiple clinical syndromes and hospital wards, rather than focusing exclusively on a single infectious syndrome or healthcare-associated infections.

## 2. Materials and Methods

This analysis was conducted prior to the implementation of our syndromic antibiogram intervention at HCM, with the aim of characterizing antibiotic prescribing practices among pediatric patients and to provide baseline evidence to inform future optimization of prescribing protocols and the development of antimicrobial stewardship (AMS) initiatives.

### 2.1. Study Design and Setting

A retrospective cross-sectional study with a quantitative approach was conducted among pediatric patients admitted to HCM between January and December 2023. HCM is the national referral hospital of Mozambique, functioning both as a teaching institution and a research center. Located in the capital city, Maputo, HCM provides comprehensive inpatient care for pediatric and adult patients. Like many healthcare settings in LMIC, it faces substantial challenges in microbiological diagnostics, which often necessitate widespread empiric antibiotic use. The pediatric department at HCM is organized into four units: the Pediatric Intensive Care Unit (PICU), the Infant Ward, the Infectious Diseases Ward (ID), and the General Diseases Ward (GD). Patients admitted to the PICU were eligible for inclusion in the study. However, because their medical records could not be accessed during the data collection period, they were not included in the present analysis.

### 2.2. Data Collection

Data were collected through a retrospective review of the medical records of pediatric patients admitted to the study wards. Eligible participants were children aged 1 month to 14 years with a physician-documented diagnosis of suspected bacterial infection recorded at admission or during hospitalization and with complete clinical records containing sociodemographic, clinical, microbiological, and antibiotic prescribing information. Patients managed exclusively in the outpatient setting, and those with incomplete clinical records (missing sociodemographic, clinical, or antibiotic prescribing data) were excluded.

For the purposes of this study, suspected bacterial infection was defined according to the treating physician’s documented clinical diagnosis recorded in the patient’s medical record. Confirmation by microbiological testing was not required for study inclusion, reflecting routine clinical practice in this setting.

Microbiologically guided therapy was defined as antibiotic treatment initiated or modified based on microbiological laboratory findings documented in the patient’s medical record, including culture results from blood, urine, or other clinical specimens when available. Empiric therapy was defined as antibiotic treatment initiated without available microbiological laboratory results at the time of prescribing.

Upper and lower respiratory tract infections were classified according to the treating physician’s documented clinical diagnosis recorded in the patients’ medical records.

Sociodemographic data extracted from the medical records included age, sex, ward of admission, anthropometric measurements (weight and height), and place of residence. Clinical variables included presenting symptoms; comorbidities; admission and discharge dates; length of hospitalization, categorized into four groups (1–5, 6–14, 15–30, and >30 days); documented clinical diagnosis; patient outcomes; and microbiological investigations requested. Data on antibiotic prescribing included previous antibiotic exposure, prescribed antimicrobial agent, dosage, route of administration, timing of initiation, and duration of therapy. Information on microbiological investigations, including specimen type, laboratory test requested, culture result (positive or negative), and documented antibiotic treatment strategy, was extracted from the patients’ medical records. Turnaround time for laboratory results and linkage of antimicrobial susceptibility results to individual antibiotic prescriptions were not available in the reviewed records.

### 2.3. Data Analysis

Nutritional status was assessed using weight-for-height/length z-scores calculated with the WHO Anthro package in R. version 4.6.0 Biologically implausible (flagged) z-score values were excluded in accordance with the WHO criteria. Wasting was defined as a z-score < −2; children meeting this threshold were classified as wasted, whereas all others were classified as non-wasted.

Descriptive statistics were used to summarize sociodemographic characteristics, clinical presentations, antibiotic prescribing patterns, bacteriological diagnostic testing, and treatment outcomes. Categorical variables are presented as frequencies and proportions, with 95% confidence intervals (CIs) for proportions calculated using the Wilson method. Continuous variables were assessed for distributional assumptions and, due to non-normal distributions, are summarized using medians with interquartile ranges (IQRs) and minimum–maximum values.

Age was analyzed both as a continuous variable and categorized into clinically meaningful pediatric age groups to account for differences in developmental stage, disease epidemiology, and antibiotic-prescribing patterns. Our data were retrospectively extracted from routine medical records of hospitalized children; the completeness of recorded information varied across variables. Consequently, the number of observations and corresponding denominators differ in our analyses.

Associations between categorical variables were initially assessed using Pearson’s chi-square test or Fisher’s exact test, as appropriate, depending on expected cell frequencies. When overall associations were statistically significant and multiple categories were compared, post hoc pairwise comparisons were performed using Fisher’s exact test with Bonferroni correction to identify specific group differences while controlling for multiple comparisons.

Length of hospital stay was analyzed as a categorical descriptive variable. For visualization purposes, hospital stay was categorized into four groups (1–5, 6–14, 15–30, and >30 days). Associations between length of hospital stay and antibiotic regimen were evaluated using Pearson’s chi-square test, followed by Bonferroni-adjusted post hoc pairwise comparisons when appropriate.

Factors associated with (i) requesting bacteriological diagnostic testing and (ii) receiving combination antibiotic therapy versus monotherapy were evaluated using logistic regression models. Univariable logistic regression analyses were first performed to estimate crude odds ratios (ORs) with 95% confidence intervals (CIs). Candidate variables for the multivariable model were selected a priori based on clinical and epidemiological relevance and/or evidence of association in univariable analyses. To ensure model parsimony and reduce the risk of overfitting, the final model included only variables considered clinically relevant and with a plausible contribution to explaining the outcome. Multivariable logistic regression models were used to estimate adjusted odds ratios (AORs) with 95% CIs. All statistical tests were two-sided, and *p*-values < 0.05 were considered statistically significant.

Where decision support is most needed, antibiotics were classified according to the 2023 World Health Organization (WHO) Access, Watch, and Reserve (AWaRe) classification at the individual drug level. For descriptive purposes, prescribed antibiotics were grouped into pharmacological classes when presenting the distribution of antibiotic use. The complete list of prescribed antibiotics, their pharmacological classes, and corresponding AWaRe categories is provided in Table A1 (Appendix A).

To visually represent clinical decision-making pathways, Sankey diagrams were generated to illustrate patient flow from diagnosis to treatment strategy, antibiotic regimen (monotherapy versus combination therapy), length of hospital stay, and clinical outcomes, thereby highlighting the interrelationships between prescribing decisions and patient trajectories. All statistical analyses were conducted using R software version 4.6.0 (R Foundation for Statistical Computing, Vienna, Austria).

## 3. Results

### 3.1. Demographic Characteristics

#### Demographic Characteristics and Clinical Symptoms Among Pediatric Patients

A total of 358 pediatric patients with suspected or confirmed bacterial infections were included in this analysis. Males accounted for 56% (*n* = 188) of the sample. The median age was 14 months (IQR: 6–48 months), with 53% aged 1–23 months, 23% aged 2–5 years, 12% aged 6–10 years, and 5.7% aged 11–14 years. Nearly half of the patients (49%) were admitted to the Infant Ward, followed by the General Diseases Ward (33%) and the Infectious Diseases Ward (18%).

The most frequently reported symptoms were fever (body temperature ≥ 38 °C), cough, diarrhea, and respiratory distress in 71%, 50%, 31%, and 28%, respectively. Among patients with recorded comorbidities (*n* = 64), wasting (z-score < −2) was the most frequent condition, affecting 34%, followed by anemia at 28% (Table 1).

### 3.2. Microbiological Investigations

A total of 134 microbiological investigations were performed among hospitalized pediatric patients. Urine specimens were the most frequently collected (71/134, 53.0%), followed by blood (55/134, 41.0%), pus (7/134, 5.2%), and cerebrospinal fluid (1/134, 0.7%).

Accordingly, the most frequently requested microbiological tests were urine cultures (68/134, 50.7%) and blood cultures (57/134, 42.5%), while pus cultures (8/134, 6.0%) and cerebrospinal fluid cultures (1/134, 0.7%) were requested less frequently. Overall, 104 of the 134 microbiological investigations (77.6%) yielded positive bacterial growth, whereas 30 (22.4%) showed no growth. Detailed results are presented in Table A2 (Appendix A).

Turnaround time for microbiological results was not available from the reviewed clinical records and therefore could not be assessed. Likewise, antimicrobial susceptibility test results could not be linked to individual antibiotic prescriptions, precluding assessment of whether antibiotic therapy was modified according to susceptibility results.

### 3.3. Clinical Pathways Cascade from Diagnosis to Outcome

**Figure 1:**
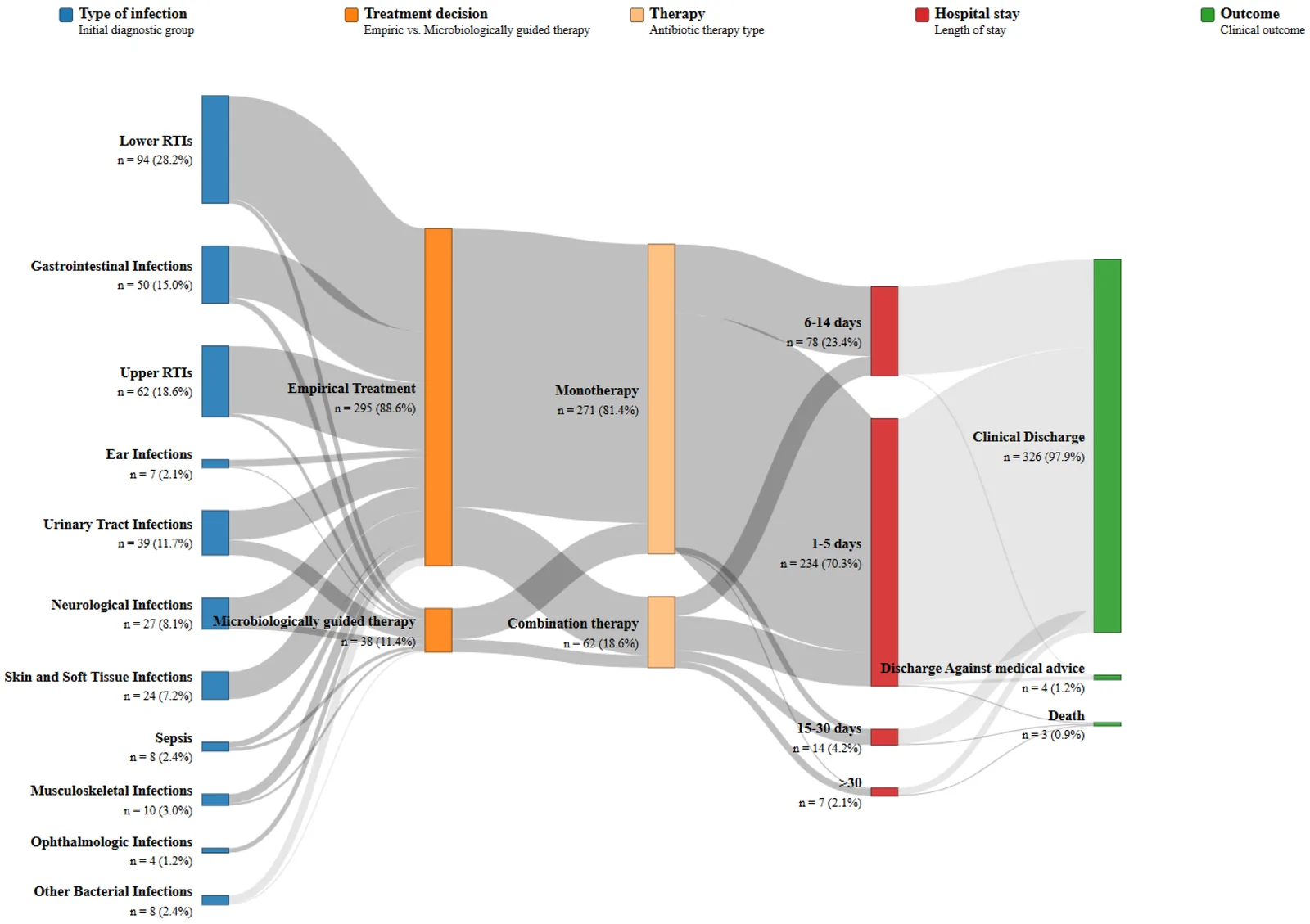
Sankey diagram of pediatric patients from diagnosis to treatment type, antibiotic therapy, length of hospital stay, and clinical outcome.

Figure 1 presents a Sankey diagram illustrating the clinical pathways of 333 hospitalized pediatric patients from diagnostic classification through treatment decisions, hospitalization duration, and clinical outcomes. The figure highlights the relationship between infection syndrome, the use of empiric versus microbiologically guided therapy, antibiotic regimen, length of stay, and final disposition. For descriptive purposes, length of hospital stay was categorized into four groups (1–5, 6–14, 15–30, and >30 days). The distribution of antibiotic regimens across these categories is presented in Figure 1.

Lower respiratory tract infections (*n* = 94, 28.2%) were the most common diagnostic category, followed by upper respiratory tract infections (*n* = 62, 18.6%), gastrointestinal infections (*n* = 57, 15.9%), and urinary tract infections (*n* = 39, 11.7%). Overall, empiric antibiotic prescribing predominated, accounting for 88.6% (*n* = 295) of antibiotic selection, whereas microbiologically guided therapy was used in only 11.4% of cases (*n* = 38). However, treatment patterns varied significantly by diagnostic category (*p* < 0.001). Compared with respiratory tract infections and skin and soft tissue infections, UTIs were substantially more likely to undergo bacteriological testing and receive microbiologically guided therapy. Following post hoc pairwise comparisons using Fisher’s exact test with Bonferroni correction demonstrated that microbiologically guided therapy was significantly more frequent among children with urinary tract infections than among those with lower respiratory tract infections (33.3% vs. 4.3%; *p* = 0.001) and upper respiratory tract infections (33.3% vs. 4.8%; *p* = 0.021).

The antibiotic regimen was also associated with the treatment approach (*p* = 0.037). Monotherapy was the most common treatment strategy, prescribed in 81.4% (*n* = 271) of cases, followed by combination therapy in 18.6% (*n* = 62). Patients receiving combination therapy were more likely to have microbiologically guided treatment than those receiving monotherapy (17.7% vs. 10.0%), whereas microbiologically guided treatment was exclusively observed among patients who received antibiotic therapy.

Length of hospitalization varied significantly according to antibiotic regimen (*p* < 0.001). Monotherapy predominated among children hospitalized for 1–5 days, whereas the proportion of combination antibiotic therapy increased progressively with longer hospital stays, accounting for 21.8% of hospitalizations lasting 6–14 days, 64.3% of those lasting 15–30 days, and 85.7% of hospitalizations exceeding 30 days. Post hoc pairwise comparisons with Bonferroni correction showed that combination antibiotic therapy was significantly more frequent among children hospitalized for 15–30 days than among those hospitalized for 1–5 days and 6–14 days. Similarly, combination therapy was significantly more frequent among children hospitalized for more than 30 days than among those hospitalized for 1–5 days and 6–14 days.

Clinical outcomes were generally favorable, with 326 of 333 patients (97.9%) discharged clinically improved. Other outcomes included discharge on the family’s request (*n* = 4) and death (*n* = 3). The association between antibiotic regimen and clinical outcome was not statistically significant (*p* = 0.110). No statistically significant association was observed between the duration of hospitalization and clinical outcome (Fisher’s exact test, *p* = 0.050).

### 3.4. Distribution of Antibiotic Classes Among Prescribed Antibiotics

The distribution of prescribed antibiotics according to the WHO AWaRe classification framework demonstrated a predominance of “Watch” group antibiotics. Overall, Watch antibiotics accounted for 61.6% (*n* = 330) of prescriptions, largely driven by the high use of cephalosporins. Access antibiotics represented 36.4% (*n* = 195) of prescriptions, including penicillins, aminoglycosides, cotrimoxazole, nitroimidazoles, anphenicols, beta-lactamase inhibitors, lincosamides, and tetracycline.

Reserve antibiotics were infrequently used, comprising 1.9% (*n* = 10) of total prescriptions, primarily carbapenems. A small proportion of antibiotics (0.2%, *n* = 1) could not be clearly classified within the AWaRe framework due to class-level aggregation, including polypeptides (Table 2).

### 3.5. Factors Associated with Microbiologically Guided Antibiotic Therapy

Microbiologically guided antibiotic therapy was more frequently prescribed among patients admitted to the infectious diseases ward, those presenting with decreased skin turgor, and those referred from another healthcare facility. In the adjusted logistic regression model, admission to the infectious diseases ward remained independently associated with higher odds of receiving microbiologically guided therapy compared with admission to the general medicine ward (AOR = 3.17, 95% CI: 1.08–9.89; *p* = 0.04). Although patients admitted to the infant ward also had higher odds of receiving microbiologically guided therapy, this association was no longer statistically significant after adjustment (AOR = 2.11, 95% CI: 0.79–6.28; *p* = 0.14).

Among the clinical characteristics evaluated, decreased skin turgor was independently associated with microbiologically guided therapy. Patients presenting with this clinical sign had approximately fourfold higher odds of receiving microbiologically guided therapy than those without decreased skin turgor (AOR = 3.72, 95% CI: 1.31–10.18; *p* = 0.01). In contrast, cough was associated with lower odds of microbiologically guided therapy in the univariable analysis (OR = 0.44, 95% CI: 0.21–0.88; *p* = 0.02), but this association was only borderline significant after adjustment (AOR = 0.47, 95% CI: 0.20–1.02; *p* = 0.05).

The referral source was also independently associated with microbiologically guided therapy. Compared with patients admitted directly from home, those referred from another hospital had nearly sixfold higher odds of receiving microbiologically guided therapy (AOR = 5.96, 95% CI: 2.24–15.54; *p* < 0.001). Likewise, intrahospital transfers were associated with significantly higher odds of microbiologically guided therapy (AOR = 12.79, 95% CI: 1.39–156.36; *p* = 0.03), although this estimate should be interpreted with caution given the small number of patients in this subgroup.

Several variables were associated with microbiologically guided therapy in the univariable analysis but did not retain statistical significance after adjustment, including seizures and admission to the infant ward. No significant associations were observed for fever, respiratory distress, diarrhea, dehydration, pallor, type of infection, or underlying comorbidities (Table 3).

### 3.6. Factors Associated with Combination Antibiotic Therapy

Combination antibiotic therapy was more frequently prescribed among children presenting with respiratory distress (26.1%, 24/92) than among those without respiratory distress (15.8%, 38/241). A higher proportion of combination therapy was also observed among children referred from another hospital (42.3%, 11/26) or transferred from another hospital department (33.3%, 1/3), compared with those admitted directly from home (16.5%, 48/291). Conversely, combination therapy was prescribed less frequently among children presenting with diarrhea (11.7%, 12/103) than among those without diarrhea (21.7%, 50/230).

In the adjusted logistic regression analysis, respiratory distress was the only factor independently associated with combination antibiotic therapy. Children presenting with respiratory distress had nearly fivefold higher odds of receiving combination therapy than those without respiratory distress (AOR = 4.79, 95% CI: 1.22–21.92; *p* = 0.024). Although interhospital referral was associated with increased odds of combination therapy in the univariable analysis, this association was no longer statistically significant after adjustment for potential confounders. Likewise, the inverse association observed for diarrhea in the univariable analysis did not remain significant in the adjusted model. Detailed results are presented in Table A3 (Appendix A).

## 4. Discussion

This study provides valuable insights into antibiotic prescribing patterns among hospitalized pediatric patients at a tertiary referral hospital in Mozambique, highlighting practices in a setting characterized by limited use of microbiological diagnostics and the absence of syndromic antibiogram-guided empiric therapy. The findings revealed a predominance of empiric antibiotic prescribing, frequent use of WHO Watch antibiotics, widespread reliance on monotherapy, and generally short hospital stays. In addition, prescribing patterns differed according to selected clinical characteristics, referral source, and hospital ward. Multivariable analyses further demonstrated that respiratory distress, decreased skin turgor, admission to the infectious diseases ward, and referral from another healthcare facility were independently associated with specific antibiotic prescribing strategies. Overall, these observations align with reports from other LMICs, where diagnostic limitations, resource constraints, and a high burden of infectious diseases continue to influence antimicrobial prescribing practices [19,24,25,26].

The overwhelming predominance of empiric treatment, observed in 89% of our hospitalized pediatric patients, is an important finding. Similar patterns have been reported across sub-Saharan Africa [27,28]. A multicenter review of antimicrobial use in African hospitals demonstrated that empiric antibiotic prescribing remains the predominant therapeutic approach in most pediatric settings due to limited access to microbiological diagnostics, delayed laboratory turnaround times, inadequate infection prevention and control measures, and suboptimal adherence to clinical guidelines [29,30]. Likewise, a point prevalence survey conducted in three Nigerian public children’s hospitals reported empiric prescribing rates exceeding 81% among neonates and 89.3% of pediatric patients, reflecting the absence of ASP [31]. Comparable findings have also been documented in Ghana and Uganda, where microbiological confirmation remains underutilized in routine pediatric care [32,33].

The predominance of empiric therapy in the current study reflects the reality in Mozambique, where clinicians are frequently required to initiate treatment before microbiological results become available. In many sub-Saharan African hospitals, laboratory infrastructure remains insufficient, with recurrent shortages of culture media, reagents, and trained personnel [5,34]. Furthermore, the urgency of initiating treatment in severely ill children often reinforces reliance on empiric broad-spectrum therapy. Although empiric antibiotic therapy is clinically appropriate in many situations, particularly among children with suspected severe bacterial infections, the high reliance on empiric prescribing observed in this study highlights the importance of strengthening access to local microbiological and antimicrobial susceptibility data to better inform empirical treatment decisions [5,35].

One of the most important findings of this study was the limited use of microbiologically guided antibiotic therapy, which was prescribed in only 11% of hospitalized children. The multivariable analysis showed that children admitted to the infectious diseases ward, those presenting with decreased skin turgor, and those referred from another healthcare facility had significantly higher odds of receiving microbiologically guided therapy. These findings suggest that microbiological investigations may have been preferentially requested for children perceived to have more complex clinical presentations or requiring closer diagnostic evaluation. Although the study design does not allow conclusions regarding clinicians’ decision-making processes, these observations indicate that the use of microbiological investigations was not uniformly distributed across patient groups.

Although microbiologically guided therapy was more frequently observed among children with urinary tract infections than among those with respiratory tract infections, this difference should be interpreted cautiously because infection type was not independently associated with microbiologically guided therapy after adjustment. The higher proportion of microbiologically guided therapy observed in urinary tract infections is likely attributable, at least in part, to the greater availability and diagnostic yield of urine cultures. In contrast, microbiological confirmation of respiratory infections in children is often limited by challenges in obtaining representative respiratory specimens and the lower sensitivity of conventional diagnostic methods, which may contribute to greater reliance on empiric antibiotic therapy. Nevertheless, the higher proportion of microbiologically guided treatment among urinary tract infections is consistent with previous studies from Ghana and other African settings, where urine cultures are more commonly requested than respiratory or blood cultures owing to the relative ease of specimen collection and higher diagnostic yield [28]. This difference may reflect the relative accessibility and diagnostic feasibility of urine sampling compared with invasive or lower-yield microbiological investigations required for respiratory syndromes. In contrast, respiratory tract infections in the present study were managed almost exclusively empirically, with more than 95% of cases receiving empiric treatment. Conversely, respiratory tract infections were managed almost exclusively with empiric therapy, reflecting current syndromic management practices widely adopted across many pediatric settings in sub-Saharan Africa [32].

Another relevant finding was the predominance of Watch antibiotics according to the WHO AWaRe classification, accounting for nearly two-thirds of prescribed antibiotics. The predominance of Watch antibiotics has important implications for AMS because these agents, particularly third-generation cephalosporins, are associated with increased selective pressure for antimicrobial resistance. Similar prescribing profiles characterized by extensive ceftriaxone use have been reported in pediatric hospitals in South Africa, Tanzania, and other sites in Mozambique [30,32,36]. For example, a recent study conducted in northern Mozambique similarly identified ceftriaxone as one of the most frequently prescribed antibiotics among hospitalized children, reflecting increasing reliance on broad-spectrum therapy within tertiary healthcare settings [19]. The extensive use of Watch antibiotics observed in the current study may therefore represent both a response to clinical severity and a consequence of limited access to local microbiological surveillance data capable of guiding narrower empirical therapy.

Despite the high frequency of empiric prescribing, monotherapy remained the predominant therapeutic strategy across most clinical categories. This finding is consistent with AMS principles, which recommend the use of the narrowest effective antibiotic regimen whenever clinically appropriate. In the adjusted analysis, respiratory distress was the only factor independently associated with combination antibiotic therapy, with affected children presenting nearly fivefold higher odds of receiving combination therapy than those without respiratory distress. Although combination therapy was also more frequently prescribed among children referred from other hospitals, this association did not remain statistically significant after adjustment.

A greater proportion of children receiving combination therapy also underwent microbiologically guided treatment, suggesting that broader antibiotic regimens were more frequently used among patients undergoing additional microbiological investigation.

Similar associations between clinical severity indicators and broader-spectrum antibiotic regimens have been described in pediatric antimicrobial utilization studies conducted in Ghana and Kenya [36,37]. Taken together, these findings suggest that combination therapy was more commonly prescribed in children presenting with features suggestive of greater clinical complexity or perceived illness severity. However, given the retrospective observational design, these associations should not be interpreted as evidence of a causal relationship between clinical characteristics and antibiotic prescribing decisions.

The findings highlight important AMS challenges in pediatric care in Mozambique. The high reliance on empiric prescribing and Watch antibiotics suggests limited integration of microbiological diagnostics and emphasizes the need for stronger AMS interventions and improved diagnostic capacity. The observed variability in microbiologically guided therapy across hospital wards and referral pathways further suggests opportunities to improve equitable access to microbiological investigations as part of future stewardship interventions.

These results should be interpreted considering some limitations. The study was conducted in a single quaternary referral hospital, which may limit generalizability to other healthcare settings. In addition, the retrospective design relied on routinely collected clinical records, resulting in variable levels of missing data across some clinical variables due to incomplete documentation. Analyses were therefore performed using the available data for each variable. Although the overall proportion of missing data was low, differential missingness may have introduced information bias and could have influenced some descriptive estimates. Although hospital microbiological and antimicrobial susceptibility data were available, these data could not be linked to individual antibiotic prescriptions. Therefore, the appropriateness of prescribing at the patient level could not be assessed. An additional limitation is the low frequency of adverse clinical outcomes observed in the study population. As most patients were discharged with clinical improvement and only a small number experienced unfavorable outcomes, the study was underpowered to evaluate associations between antibiotic treatment strategies and clinical outcomes. Consequently, the outcome analyses should be interpreted as descriptive rather than inferential, and no causal conclusions regarding the relationship between antibiotic prescribing practices and patient outcomes can be drawn.

Furthermore, information regarding laboratory turnaround time was not available in the reviewed clinical records and therefore could not be evaluated, limiting assessment of its potential influence on antibiotic prescribing decisions. Another limitation is that patients admitted to the Pediatric Intensive Care Unit were not included because access to their medical records was not available during the study period. Therefore, the findings may not be generalizable to critically ill pediatric patients. Finally, some subgroup analyses included small sample sizes, potentially affecting the precision of certain estimates.

Despite these limitations, the study provides important baseline evidence on pediatric antibiotic prescribing practices in Mozambique and addresses a key evidence gap in AMS in sub-Saharan Africa. The findings emphasize the need to strengthen microbiological diagnostic capacity, improve access to culture and susceptibility testing, and expand antimicrobial surveillance systems to guide empiric therapy better. The implementation of locally adapted syndromic antibiograms, alongside continuous stewardship training, prescription audits, and feedback mechanisms, may help support evidence-informed empirical antibiotic selection and strengthen antimicrobial stewardship activities. Future prospective multicenter studies incorporating microbiological and susceptibility data are needed to further evaluate the appropriateness of empirical therapy and support context-specific stewardship interventions.

## 5. Conclusions

This study provides baseline evidence on antibiotic prescribing practices among hospitalized pediatric patients before implementation of a syndromic antibiogram intervention at a tertiary referral hospital in Mozambique. The predominance of empiric antibiotic therapy, limited use of microbiologically guided treatment, and frequent prescription of WHO Watch antibiotics characterize current prescribing practices and highlight opportunities to strengthen antimicrobial stewardship through enhanced microbiological diagnostic capacity and locally generated antimicrobial susceptibility data. These findings establish an important pre-implementation benchmark and provide the contextual evidence required to support the implementation and future evaluation of a locally adapted syndromic antibiogram in this setting.

## Figures and Tables

**Table 1:** Demographic characteristics and clinical symptoms among pediatric patients with bacterial infections at Hospital Central de Maputo.

Characteristics	%	*n*/N
Sex		
Male	56%	*188*/333
Female	44%	*145*/333
Age in Months [Median (Q1–Q3), Min–Max]	14 (6–48) 1–168	
Age group		
1–23 months	53%	*196*/333
2–5 years	23%	*77*/333
6–10 years	12%	*41*/333
11–14 years	5.7%	*19*/333
Ward		
Infant ward	49%	*162*/333
Infectious Diseases ward	18%	*61*/333
General Diseases ward	33%	*110*/333
Observed symptoms		
Cough	50%	*166*/333
Fever (body temperature ≥ 38 °C)	71%	*250*/332
Respiratory distress	28%	*92*/333
Diarrhea	31%	*103*/333
Seizures	12%	*40*/331
Dehydration	18%	*59*/326
Pallor	1.8%	*6*/333
Decreased skin turgor	7.2%	*24*/333
Source of Referral		
From home	91%	*291*/320
Interhospital transfer	8.1%	*26*/320
Intrahospital transfer	0.9%	*3*/320
Morbidities		
Anemia	28%	*17*/60
Dermatitis	5%	*3*/60
Wasting (z-score < −2)	34%	*21*/60
Congenital diseases	3.3%	*2*/60
Respiratory infections	6.7%	*4*/60
Parasitism	15%	*9*/60
Other ^a^	6.7%	*4*/60

^a^ Other includes neurological sequelae, renal insufficiency, and allergic syndrome.

**Table 2:** Distribution of antibiotic prescriptions according to the WHO AWaRe classification.

AWaRe Category	Antibiotic Classes Included	N (%)
Access	Penicillins, Aminoglycosides, Cotrimoxazole, Nitroimidazoles, Amphenicols, Beta-lactamase inhibitors, Lincosamides, Tetracycline	195 (36.4)
Watch	Cephalosporins, Fluoroquinolones, Macrolides, Glycopeptides, Beta-lactamase inhibitors	330 (61.6)
Reserve	Carbapenems	10 (1.9)
Unclassified	Polypeptides	1 (0.2)
Total	-	536 (100)

**Table 3:** Factors associated with requesting bacteriological diagnostic testing among hospitalized children: univariable and multivariable logistic regression analysis.

Characteristics	Categories	% (*n*/N)	OR (95% CI)	*p*-Value	AOR (95% CI)	*p*-Value
Ward	General medicine ward	5.5% (6/110)	Reference			
	Infectious diseases ward	18% (11/61)	4.03 (1.49–11.76)	0.006	3.17 (1.08–9.89)	0.04
	Infant ward	13% (21/162)	2.64 (1.12–7.11)	0.026	2.11 (0.79–6.28)	0.14
Cough	No	16% (26/167)	Reference			
	Yes	7.2% (12/166)	0.44 (0.21–0.88)	0.02	0.47 (0.20–1.02)	0.05
Fever (body temperature ≥ 38 °C)	No	10% (10/97)	Reference			
	Yes	12% (28/235)	1.18 (0.58–2.62)	0.65		
Respiratory distress	No	12% (29/241)	Reference			
	Yes	9.8% (9/92)	0.86 (0.38–1.80)	0.7		
Diarrhea	No	9.1% (21/230)	Reference			
	Yes	17% (17/103)	1.92 (0.97–3.77)	0.06		
Seizures	No	10% (29/291)	Reference			
	Yes	23% (9/40)	2.49 (1.06–5.44)	0.036	2.12 (0.73–5.76)	0.16
Dehydration	No	10% (27/267)	Reference			
	Yes	15% (9/59)	1.77 (0.76–3.80)	0.18		
Pallor	No	11% (37/327)	Reference			
	Yes	17% (1/6)	2.29 (0.23–11.93)	0.41		
Decreased skin turgor	No	9.1% (28/309)	Reference			
	Yes	42% (10/24)	7.79 (3.16–18.78)	*p* < 0.001	3.72 (1.31–10.18)	0.01
Provenance (Source of Referral)	House	7.9% (23/291)	Reference			
	Interhospital transfer	42% (11/26)	8.15 (3.41–19.12)	*p* < 0.001	5.96 (2.24–15.54)	<0.001
	Intrahospital transfer	67% (2/3)	20.67 (2.64–232.83)	0.01	12.79 (1.39–156.36)	0.03
Type of infection	Lower RTIs	4.3% (4/94)	0.22 (0.03–2.38)	0.179		
	Gastrointestinal Infections	10% (5/50)	0.52 (0.08–5.69)	0.541		
	Upper RTIs	4.8% (3/62)	0.25 (0.03–2.92)	0.234		
	Ear Infections	14% (1/7)	Reference			
	Urinary Tract Infections	33% (13/39)	2.21 (0.40–22.76)	0.385		
	Neurological Infections	22% (6/27)	1.31 (0.21–14.24)	0.786		
	Skin and Soft Tissue Infections	0% (0/24)	0.09 (0.0006–1.86)	0.116		
	Musculoskeletal Infections	20% (2/10)	1.27 (0.13–16.53)	0.833		
	Ophthalmologic Infections	0% (0/4)	0.48 (0.00–11.39)	0.66		
	Sepsis	38% (3/8)	2.76 (0.33–35.41)	0.357		
	Other Bacterial Infections	13% (1/8)	1.26 (0.18–14.38)	0.83		
Morbidities	Anemia	35% (6/17)	Reference			
	Dermatitis	0% (0/3)	0.25 (0.002–3.26)	0.327		
	Congenital diseases	0% (0/2)	0.35 (0.002–5.30)	0.487		
	Respiratory infections	0% (0/4)	0.20 (0.001–2.34)	0.225		
	Parasitism	11% (1/9)	0.31 (0.03–1.92)	0.22		
	Wasting (z-score < −2)	14% (3/21)	0.33 (0.07–1.43)	0.141		
	Other	75% (3/4)	4.13 (0.54–49.97)	0.175		

## Data Availability

The deidentified data supporting this study are available at the Open Science Framework (OSF) repository and can be accessed via the following link: https://osf.io/r2kw4/ (accessed on 27 July 2026). Data access was authorized by the Chief of the Pediatric Department, Dr. Eugénia Macassa.

## Abbreviations

The following abbreviations are used in this manuscript:

AWaRe	Access, Watch, and Reserve
AMR	Antimicrobial resistance
ASPs	Antimicrobial stewardship programs
CI	Confidence interval
GD	General Diseases Ward
HCM	*Hospital Central de Maputo*
ICU	Intensive Care Unit
ID	Infectious Diseases Ward
LMI	Low- and Middle-Income Countries
PICU	Pediatric Intensive Care Unit
UTI	Urinary tract infection
WHO	World Health Organization

## Appendix A

**Table A1:** Antibiotics.

Antibiotic Name	Pharmacological Class	WHO AWaRe Category	*n*	%
Crystallized penicillin	Penicillins	Access	94	17.5%
Ampicillin	Penicillins	Access	23	4.3%
Amoxicillin	Penicillins	Access	3	0.6%
Ceftriaxone	Third-generation-cephalosporins	Watch	286	53.4%
Cefotaxime	Third-generation-cephalosporins	Watch	1	0.2%
Gentamicin	Aminoglycosides	Access	48	9.0%
Amikacin	Aminoglycosides	Access	1	0.2%
Sulfamethoxazole/trimethoprim	Sulfonamide-trimethoprim-combinations	Access	9	1.7%
Imipenem	Carbapenems	Reserve	10	1.9%
Ciprofloxacin	Fluoroquinolones	Watch	14	2.6%
Piperacillin/tazobactam	Beta-lactam/beta-lactamase-inhibitor_anti-pseudomonal	Watch	4	0.7%
Amoxicillin/clavulanic-acid	Beta-lactam/beta-lactamase-inhibitor	Access	1	0.2%
Chloramphenicol	Amphenicols	Access	2	0.4%
Tetracycline	Tetracyclines	Access	1	0.2%
Vancomycin_IV	Glycopeptides	Watch	13	2.4%
Bacitracin	Polypeptides	None	1	0.2%
Azithromycin	Macrolides	Watch	11	2.1%
Erythromycin	Macrolides	Watch	1	0.2%
Metronidazole	Imidazoles	Access	11	2.1%
Clindamycin	Lincosamides	Access	2	0.4%
Total			536	100.0%

**Table A2:** Microbiology investigation.

Characteristic	% (*n*/N)
Sample type	
Cerebrospinal Fluid	0.7% (1/134)
Pus	5.2% (7/134)
Blood	41% (55/134)
Urine	53% (71/134)
Type of laboratory test	
Pus Culture	6% (8/134)
Urine culture	50.7% (68/134)
Blood culture	42.5% (57/134)
Cerebrospinal fluid culture	0.7% (1/134)
Subculture	
Negative	22.4% (30/134)
Positive	77.6% (104/134)

**Table A3:** Clinical and epidemiological factors associated with antibiotic monotherapy versus combination therapy among hospitalized children: univariable and multivariable logistic regression analyses.

Characteristics	Categories	% (*n*/N)	OR (95% CI)	*p*-Value	AOR (95% CI)	*p*-Value
Ward	General medicine ward	22% (24/110)	Reference			
	Infectious diseases ward	13% (8/61)	0.56 (0.23–1.27)	0.171		
	Infant ward	19% (30/162)	0.81 (0.45–1.48)	0.497		
Cough	No	19% (32/167)	Reference			
	Yes	18% (30/166)	0.93 (0.54–1.61)	0.8		
Fever (body temperature ≥ 38 °C)	No	13% (13/97)	Reference			
	Yes	20% (48/235)	1.62 (0.86–3.22)	0.138		
Respiratory distress	No	16% (38/241)	Reference			
	Yes	26% (24/92)	1.89 (1.05–3.35)	0.033	4.79 (1.22–21.92)	0.024
Diarrhea	No	22% (50/230)	Reference			
	Yes	12% (12/103)	0.49 (0.24–0.93)	0.028	0.61 (0.13–2.53)	0.493
Seizures	No	18% (52/291)	Reference			
	Yes	23% (9/40)	1.38 (0.60–2.92)	0.435		
Dehydration	No	21% (55/267)	Reference			
	Yes	10% (6/59)	0.47 (0.18–1.04)	0.062		
Pallor	No	18% (60/327)	Reference			
	Yes	33% (2/6)	2.46 (0.42–11.36)	0.287		
Decreased skin turgor	No	18% (57/309)	Reference			
	Yes	21% (5/24)	1.24 (0.42–3.14)	0.677		
Provenance (Source of Referral)	House	16% (48/291)	Reference			
	Interhospital transfer	42% (11/26)	3.72 (1.61–8.45)	0.003	7.37 (0.88–93.32)	0.066
	Intrahospital transfer	33% (1/3)	3.01 (0.27–23.15)	0.321	0.36 (0.001–34.82)	0.648
Type of infection	Lower RTIs	19% (18/94)	1.05 (0.20–10.50)	0.961		
	Gastrointestinal Infections	14% (7/50)	0.75 (0.13–7.90)	0.773		
	Upper RTIs	18% (11/62)	0.97 (0.18–9.93)	0.973		
	Ear Infections	14% (1/7)	Reference			
	Urinary Tract Infections	15% (6/39)	0.84 (0.14–9.04)	0.864		
	Neurological Infections	19% (5/27)	1.06 (0.16–11.68)	0.955		
	Skin and Soft Tissue Infections	17% (4/24)	0.95 (0.14–10.76)	0.962		
	Musculoskeletal Infections	30% (3/10)	2.02 (0.25–25.06)	0.518		
	Ophthalmologic Infections	0% (0/4)	0.48 (0.003–11.39)	0.663		
	Sepsis	63% (5/8)	6.81 (0.86–89.80)	0.071		
	Other Bacterial Infections	25% (2/8)	1.67 (0.17–22.17)	0.661		
Morbidities	Anemia	35% (6/17)	Reference			
	Dermatitis	0% (0/3)	0.25 (0.002–3.26)	0.327	0.652 (0.25 - 1.61)	0.357
	Congenital diseases	50% (1/2)	1.77 (0.12–25.47)	0.65	2.86 (0.13–63.08)	0.488
	Respiratory infections	0% (0/4)	0.20 (0.001–2.34)	0.225	0.24 (0.001–4.32)	0.373
	Parasitism	0% (0/9)	0.09 (0.0007–0.97)	0.046	0.15 (0.001–2.19)	0.182
	Wasting (z-score < −2)	29% (6/21)	0.74 (0.19–2.83)	0.66	1.10 (0.22–5.81)	0.91
	Other	25% (1/4)	0.76 (0.06–5.93)	0.798	1.22 (0.05–17.38)	0.889
